# 2-Bromo-4-chloro-6-[(1-phenyl­ethyl)­imino­meth­yl]phenol

**DOI:** 10.1107/S1600536808031383

**Published:** 2008-10-09

**Authors:** Xinli Zhang

**Affiliations:** a Department of Chemistry, Baoji University of Arts and Science, Baoji, Shaanxi 721007, People’s Republic of China

## Abstract

The title compound, C_15_H_13_BrClNO, is a Schiff base derived from the condensation of equimolar quanti­ties of 3-bromo-5-chloro­salicylaldehyde and 1-phenyl­ethanamine. The structure displays a *trans* configuration with respect to the imine C=N double bond. The N atom is also involved in an intra­molecular O—H—N hydrogen bond, which stabilizes the configuration of the compound.

## Related literature

Schiff base ligands have demonstrated significant biological activities and new examples are being tested for their antimicrobial activity (Ali *et al.*, 2002 ▶; Cukurovali *et al.*, 2002 ▶) and antiviral activity (Tarafder *et al.*, 2002 ▶).
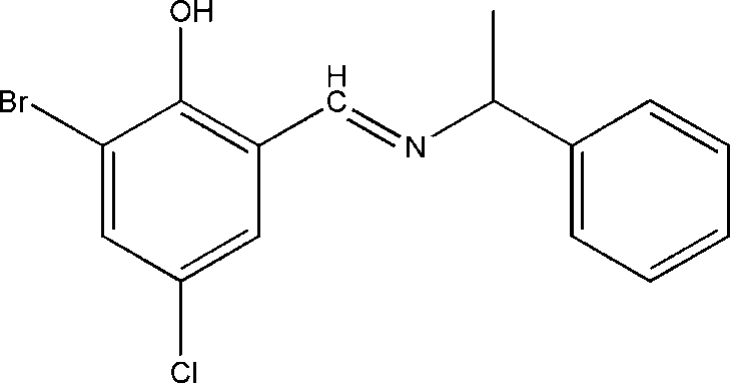


## Experimental

### 

#### Crystal data

C_15_H_13_BrClNO
*M* *_r_* = 338.62
Monoclinic, 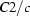

*a* = 21.764 (2) Å
*b* = 9.5088 (13) Å
*c* = 15.3591 (16) Å
β = 113.426 (2)°
*V* = 2916.6 (6) Å^3^
*Z* = 8
Mo *K*α radiation
μ = 2.99 mm^−1^
*T* = 298 (2) K
0.36 × 0.22 × 0.19 mm

#### Data collection

Bruker SMART CCD area-detector diffractometer
Absorption correction: multi-scan (*SADABS*; Sheldrick, 1996 ▶) *T*_min_ = 0.412, *T*_max_ = 0.600 (expected range = 0.389–0.566)
7192 measured reflections
2574 independent reflections
1377 reflections with *I* > 2σ(*I*)
*R*_int_ = 0.038

#### Refinement

*R*[*F* ^2^ > 2σ(*F* ^2^)] = 0.040
*wR*(*F* ^2^) = 0.108
*S* = 1.00
2574 reflections
172 parameters
H-atom parameters constrained
Δρ_max_ = 0.41 e Å^−3^
Δρ_min_ = −0.37 e Å^−3^

### 

Data collection: *SMART* (Bruker, 2000 ▶); cell refinement: *SAINT* (Bruker, 2000 ▶); data reduction: *SAINT*; program(s) used to solve structure: *SHELXS97* (Sheldrick, 2008 ▶); program(s) used to refine structure: *SHELXL97* (Sheldrick, 2008 ▶); molecular graphics: *SHELXTL* (Sheldrick, 2008 ▶); software used to prepare material for publication: *SHELXTL*.

## Supplementary Material

Crystal structure: contains datablocks I, global. DOI: 10.1107/S1600536808031383/gw2049sup1.cif

Structure factors: contains datablocks I. DOI: 10.1107/S1600536808031383/gw2049Isup2.hkl

Additional supplementary materials:  crystallographic information; 3D view; checkCIF report

## Figures and Tables

**Table 1:** Hydrogen-bond geometry (Å, °)

*D*—H⋯*A*	*D*—H	H⋯*A*	*D*⋯*A*	*D*—H⋯*A*
O1—H1⋯N1	0.82	1.87	2.591 (4)	147

**Fig. 1.**
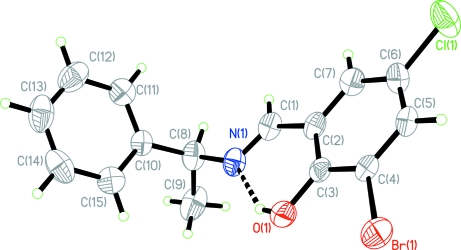
The structure of the title compound in 30% probability ellipsoids. H atoms are shown as spheres of arbitrary radii. The dotted line represent a hydrogen bond.

**Fig. 2.**
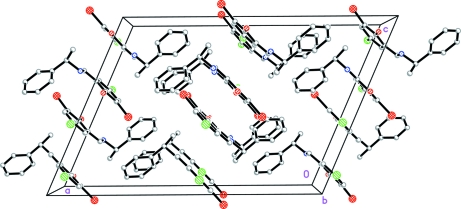
The molecular packing of (I) viewed along the b axis.

## supplementary crystallographic information

### Comment

In recent years, the role of Schiff base and its derivatives in biological processes have become a topic of study. Schiff base ligands have demonstrated significant biological activities and new examples are being tested for their antitumor, antimicrobial and antiviral activities (Tarafder *et al.*, 2002; Cukurovali *et al.*, 2002; Ali *et al.*, 2002). These properties stimulated our interest in this field. Crystals of the title compound, (I), were obtained as a new Schiff base compound. The title compound (I) is an 3-bromine-5-chloro-salicylaldehyde derivative. All bond lengths and bond angles are in the normal ranges and comparable to those observed in a similar salicylaldehyde Schiff base. its molecular Structure and a Crystal packing are illusrated in Figs. 1 and 2, respectively. The C1═N1 bond length of 1.257 (5) Å conforms to the value for a double bond. The torsional angles of C8—N1–2 and N1—C1—C2—C7 are 176.5 (4)° and -175.6 (4)°, respectively. Atom O1 deviates from the benzene mean plane by 0.026 (3)°, whereas atoms Br and Cl by 0.072 (4)° and 0.047 (4)°, respectively. The molecular structure adopts a *trans* configuration about the C1═N1 bond. In the molecule, there exists a intramolecular O—H—N hydrogen bond involving hydroxy atom O1 and imine atom N1 (Table 1). Furthermore, a more interesting phenomenon observed is shown in Fig. 2. Pairs of phen ligands from neighbouring complexes are interleaved to form a pi–pi stacking along the *c* axis. The distance between two phen ring Centroids are 3.744 (3) Å indicating significant pi–pi stacking packing interactions. The structure of (I) is thus stabilized by the hydrogen-bond system and aromatic-ring stacking interactions.

### Experimental

3-bromine-5-Chlorosalicylaldehyde (0.1 mmol, 23.55 mg) and 1-phenylethanamine (0.1 mmol, 12.1 mg) were dissolved in methanol (10 ml). The mixture was stirred for 30 min at room temperature to give a clear brown solution. After allowing the resulting solution to stand in air for 7 d, yellow block-shaped crystals of (I) were formed on slow evaporation of the solvent. The crystals were collected, washed with methanol and dried in a vacuum desiccator using anhydrous CaCl_2_ (yield 54%). Analysis found: C 46.32%, H 3.35%, calculated for C_15_H_13_Br_Cl_N_O_: C 46.33%, H 3.35%.

### Refinement

All H atoms were placed in geometrically idealized positions and constrained to ride on their parent atoms with C—H distances in the range 0.93–0.97 Å and *U*_iso_(H) = 1.2*U*_eq_ or 1.5*U*_eq_(C/O)

### Crystal data

**Table:**

C_15_H_13_BrClNO	*F*(000) = 1360
*M**_r_* = 338.62	*D*_x_ = 1.542 Mg m^−^^3^
Monoclinic, *C*2/*c*	Mo *K*α radiation, λ = 0.71073 Å
*a* = 21.764 (2) Å	Cell parameters from 1447 reflections
*b* = 9.5088 (13) Å	θ = 2.4–21.8°
*c* = 15.3591 (16) Å	µ = 2.99 mm^−^^1^
β = 113.426 (2)°	*T* = 298 K
*V* = 2916.6 (6) Å^3^	Block, yellow
*Z* = 8	0.36 × 0.22 × 0.19 mm

### Data collection

**Table:**

Bruker SMART CCD area-detector diffractometer	2574 independent reflections
Radiation source: fine-focus sealed tube	1377 reflections with *I* > 2σ(*I*)
graphite	*R*_int_ = 0.038
φ and ω scans	θ_max_ = 25.0°, θ_min_ = 2.0°
Absorption correction: multi-scan (SADABS; Sheldrick, 1996)	*h* = −25→25
*T*_min_ = 0.412, *T*_max_ = 0.600	*k* = −9→11
7192 measured reflections	*l* = −17→18

### Refinement

**Table:**

Refinement on *F*^2^	Primary atom site location: structure-invariant direct methods
Least-squares matrix: full	Secondary atom site location: difference Fourier map
*R*[*F*^2^ > 2σ(*F*^2^)] = 0.040	Hydrogen site location: inferred from neighbouring sites
*wR*(*F*^2^) = 0.108	H-atom parameters constrained
*S* = 1.00	*w* = 1/[σ^2^(*F*_o_^2^) + (0.0381*P*)^2^ + 4.3819*P*] where *P* = (*F*_o_^2^ + 2*F*_c_^2^)/3
2574 reflections	(Δ/σ)_max_ = 0.002
172 parameters	Δρ_max_ = 0.41 e Å^−^^3^
0 restraints	Δρ_min_ = −0.37 e Å^−^^3^

### Special details

**Table:**

Geometry. All e.s.d.'s (except the e.s.d. in the dihedral angle between two l.s. planes) are estimated using the full covariance matrix. The cell e.s.d.'s are taken into account individually in the estimation of e.s.d.'s in distances, angles and torsion angles; correlations between e.s.d.'s in cell parameters are only used when they are defined by crystal symmetry. An approximate (isotropic) treatment of cell e.s.d.'s is used for estimating e.s.d.'s involving l.s. planes.
Refinement. Refinement of *F*^2^ against ALL reflections. The weighted *R*-factor *wR* and goodness of fit *S* are based on *F*^2^, conventional *R*-factors *R* are based on *F*, with *F* set to zero for negative *F*^2^. The threshold expression of *F*^2^ > σ(*F*^2^) is used only for calculating *R*-factors(gt) *etc*. and is not relevant to the choice of reflections for refinement. *R*-factors based on *F*^2^ are statistically about twice as large as those based on *F*, and *R*- factors based on ALL data will be even larger.

### Fractional atomic coordinates and isotropic or equivalent isotropic displacement parameters (Å^2^)

**Table:**

	*x*	*y*	*z*	*U*_iso_*/*U*_eq_	
Br1	1.16005 (2)	0.40337 (6)	0.56363 (4)	0.0873 (3)	
Cl1	1.03028 (8)	0.89286 (15)	0.39942 (12)	0.1095 (6)	
N1	0.91088 (17)	0.2822 (5)	0.2946 (2)	0.0644 (11)	
O1	1.03226 (14)	0.2790 (3)	0.4226 (2)	0.0702 (9)	
H1	0.9971	0.2461	0.3842	0.105*	
C1	0.9157 (2)	0.4134 (6)	0.2895 (3)	0.0638 (13)	
H1A	0.8799	0.4629	0.2457	0.077*	
C2	0.9755 (2)	0.4910 (5)	0.3496 (3)	0.0554 (12)	
C3	1.0310 (2)	0.4192 (5)	0.4141 (3)	0.0526 (11)	
C4	1.08586 (19)	0.4972 (5)	0.4726 (3)	0.0545 (11)	
C5	1.0864 (2)	0.6413 (5)	0.4677 (3)	0.0583 (12)	
H5	1.1236	0.6919	0.5071	0.070*	
C6	1.0313 (2)	0.7100 (5)	0.4038 (3)	0.0646 (13)	
C7	0.9767 (2)	0.6361 (5)	0.3458 (3)	0.0662 (13)	
H7	0.9397	0.6840	0.3031	0.079*	
C8	0.8465 (2)	0.2157 (5)	0.2340 (3)	0.0716 (15)	
H8	0.8179	0.2867	0.1903	0.086*	
C9	0.8613 (3)	0.1015 (6)	0.1769 (4)	0.101 (2)	
H9A	0.8795	0.1429	0.1352	0.151*	
H9B	0.8208	0.0522	0.1401	0.151*	
H9C	0.8932	0.0369	0.2191	0.151*	
C10	0.8132 (2)	0.1667 (5)	0.2970 (3)	0.0535 (12)	
C11	0.7589 (3)	0.2392 (6)	0.2985 (3)	0.0720 (14)	
H11	0.7422	0.3159	0.2585	0.086*	
C12	0.7289 (3)	0.1996 (8)	0.3585 (5)	0.0954 (19)	
H12	0.6926	0.2505	0.3592	0.114*	
C13	0.7519 (4)	0.0872 (8)	0.4165 (4)	0.096 (2)	
H13	0.7311	0.0603	0.4562	0.115*	
C14	0.8051 (4)	0.0142 (6)	0.4165 (4)	0.0885 (18)	
H14	0.8213	−0.0624	0.4569	0.106*	
C15	0.8358 (3)	0.0533 (6)	0.3563 (4)	0.0729 (14)	
H15	0.8721	0.0019	0.3562	0.088*	

### Atomic displacement parameters (Å^2^)

**Table:**

	*U*^11^	*U*^22^	*U*^33^	*U*^12^	*U*^13^	*U*^23^
Br1	0.0491 (3)	0.0926 (4)	0.1028 (5)	0.0052 (3)	0.0120 (3)	0.0109 (3)
Cl1	0.0987 (11)	0.0682 (9)	0.1278 (14)	−0.0118 (8)	0.0094 (9)	0.0236 (9)
N1	0.060 (2)	0.077 (3)	0.054 (2)	−0.018 (2)	0.0212 (19)	−0.011 (2)
O1	0.0555 (19)	0.068 (2)	0.084 (2)	−0.0020 (16)	0.0243 (17)	−0.0038 (18)
C1	0.054 (3)	0.087 (4)	0.047 (3)	−0.008 (3)	0.016 (2)	0.003 (3)
C2	0.048 (3)	0.080 (4)	0.040 (3)	−0.007 (2)	0.020 (2)	0.000 (2)
C3	0.056 (3)	0.060 (3)	0.056 (3)	−0.005 (3)	0.037 (2)	−0.004 (2)
C4	0.042 (2)	0.070 (3)	0.055 (3)	−0.001 (2)	0.023 (2)	0.001 (2)
C5	0.052 (3)	0.073 (3)	0.052 (3)	−0.016 (2)	0.023 (2)	−0.003 (2)
C6	0.063 (3)	0.069 (3)	0.059 (3)	−0.014 (3)	0.020 (3)	0.006 (3)
C7	0.061 (3)	0.077 (4)	0.056 (3)	0.002 (3)	0.018 (2)	0.015 (3)
C8	0.062 (3)	0.092 (4)	0.052 (3)	−0.021 (3)	0.012 (3)	−0.005 (3)
C9	0.096 (4)	0.142 (5)	0.079 (4)	−0.043 (4)	0.050 (3)	−0.052 (4)
C10	0.051 (3)	0.064 (3)	0.038 (2)	−0.012 (2)	0.010 (2)	−0.006 (2)
C11	0.064 (3)	0.077 (4)	0.061 (3)	−0.006 (3)	0.010 (3)	−0.004 (3)
C12	0.067 (4)	0.120 (6)	0.100 (5)	−0.015 (4)	0.035 (4)	−0.037 (4)
C13	0.108 (5)	0.113 (6)	0.076 (4)	−0.046 (5)	0.047 (4)	−0.023 (4)
C14	0.126 (5)	0.066 (4)	0.060 (4)	−0.021 (4)	0.023 (4)	−0.003 (3)
C15	0.078 (3)	0.078 (4)	0.060 (3)	−0.002 (3)	0.024 (3)	−0.009 (3)

### Geometric parameters (Å, °)

**Table:**

Br1—C4	1.888 (4)	C8—C9	1.510 (7)
Cl1—C6	1.740 (5)	C8—H8	0.9800
N1—C1	1.257 (5)	C9—H9A	0.9600
N1—C8	1.481 (5)	C9—H9B	0.9600
O1—C3	1.339 (5)	C9—H9C	0.9600
O1—H1	0.8200	C10—C15	1.371 (6)
C1—C2	1.462 (6)	C10—C11	1.377 (6)
C1—H1A	0.9300	C11—C12	1.375 (7)
C2—C7	1.382 (6)	C11—H11	0.9300
C2—C3	1.398 (6)	C12—C13	1.354 (8)
C3—C4	1.389 (5)	C12—H12	0.9300
C4—C5	1.372 (6)	C13—C14	1.350 (8)
C5—C6	1.376 (6)	C13—H13	0.9300
C5—H5	0.9300	C14—C15	1.390 (8)
C6—C7	1.363 (6)	C14—H14	0.9300
C7—H7	0.9300	C15—H15	0.9300
C8—C10	1.494 (6)		
			
C1—N1—C8	117.8 (4)	C10—C8—H8	108.7
C3—O1—H1	109.5	C9—C8—H8	108.7
N1—C1—C2	122.5 (4)	C8—C9—H9A	109.5
N1—C1—H1A	118.7	C8—C9—H9B	109.5
C2—C1—H1A	118.7	H9A—C9—H9B	109.5
C7—C2—C3	119.5 (4)	C8—C9—H9C	109.5
C7—C2—C1	120.3 (4)	H9A—C9—H9C	109.5
C3—C2—C1	120.2 (4)	H9B—C9—H9C	109.5
O1—C3—C4	119.3 (4)	C15—C10—C11	117.8 (5)
O1—C3—C2	122.3 (4)	C15—C10—C8	122.5 (5)
C4—C3—C2	118.4 (4)	C11—C10—C8	119.7 (5)
C5—C4—C3	121.4 (4)	C12—C11—C10	120.9 (5)
C5—C4—Br1	119.3 (3)	C12—C11—H11	119.5
C3—C4—Br1	119.3 (4)	C10—C11—H11	119.5
C4—C5—C6	119.3 (4)	C13—C12—C11	120.4 (6)
C4—C5—H5	120.4	C13—C12—H12	119.8
C6—C5—H5	120.4	C11—C12—H12	119.8
C7—C6—C5	120.5 (4)	C14—C13—C12	119.9 (6)
C7—C6—Cl1	119.7 (4)	C14—C13—H13	120.0
C5—C6—Cl1	119.7 (4)	C12—C13—H13	120.0
C6—C7—C2	120.9 (4)	C13—C14—C15	120.1 (6)
C6—C7—H7	119.6	C13—C14—H14	120.0
C2—C7—H7	119.6	C15—C14—H14	120.0
N1—C8—C10	107.9 (3)	C10—C15—C14	120.8 (5)
N1—C8—C9	107.7 (4)	C10—C15—H15	119.6
C10—C8—C9	115.0 (4)	C14—C15—H15	119.6
N1—C8—H8	108.7		
			
C8—N1—C1—C2	176.5 (4)	C3—C2—C7—C6	0.3 (7)
N1—C1—C2—C7	−175.6 (4)	C1—C2—C7—C6	177.8 (4)
N1—C1—C2—C3	1.9 (7)	C1—N1—C8—C10	−109.5 (5)
C7—C2—C3—O1	178.5 (4)	C1—N1—C8—C9	125.8 (5)
C1—C2—C3—O1	1.0 (6)	N1—C8—C10—C15	−72.1 (6)
C7—C2—C3—C4	0.0 (6)	C9—C8—C10—C15	48.1 (6)
C1—C2—C3—C4	−177.6 (4)	N1—C8—C10—C11	106.0 (5)
O1—C3—C4—C5	−178.8 (4)	C9—C8—C10—C11	−133.8 (5)
C2—C3—C4—C5	−0.3 (6)	C15—C10—C11—C12	0.7 (7)
O1—C3—C4—Br1	−1.0 (5)	C8—C10—C11—C12	−177.5 (4)
C2—C3—C4—Br1	177.6 (3)	C10—C11—C12—C13	−0.8 (8)
C3—C4—C5—C6	0.3 (7)	C11—C12—C13—C14	0.8 (8)
Br1—C4—C5—C6	−177.5 (3)	C12—C13—C14—C15	−0.8 (8)
C4—C5—C6—C7	−0.1 (7)	C11—C10—C15—C14	−0.7 (7)
C4—C5—C6—Cl1	178.0 (3)	C8—C10—C15—C14	177.5 (4)
C5—C6—C7—C2	−0.2 (7)	C13—C14—C15—C10	0.7 (8)
Cl1—C6—C7—C2	−178.3 (4)		

### Hydrogen-bond geometry (Å, °)

**Table:**

*D*—H···*A*	*D*—H	H···*A*	*D*···*A*	*D*—H···*A*
O1—H1···N1	0.82	1.87	2.591 (4)	147
